# Response of diatom assemblages to the disruption of the running water continuum in urban areas, and its consequences on bioassessment

**DOI:** 10.7717/peerj.12457

**Published:** 2021-11-23

**Authors:** Ewelina Szczepocka, Paulina Nowicka-Krawczyk, Rafał M. Olszyński, Joanna Żelazna-Wieczorek

**Affiliations:** Faculty of Biology and Environmental Protection, Department of Algology and Mycology, University of Lodz, Łódź, Poland

**Keywords:** Diatom assemblages, Bioassessment, Diatom indices, Hydromorphological transformations, Urban streams, Ponds

## Abstract

Transformation of river and stream channels disrupts their natural ecological cycles and interrupts the continuum of their ecosystems. Changes in natural hydromorphological conditions transform lotic communities into those atypical of flowing waters, resulting in bioassessment procedures yielding incorrect results. This study shows how hydromorphological transformations of ecosystems affect the ecological status bioassessment results by disturbing diatom communities typical for rivers. Moreover, the article presents a new biological assessment procedure for urban transformed rivers including the verification of the community structure based on autecology and quantity of species. The ecological status of the ecosystem was assessed using benthic diatom assemblages and supported with results of hydrochemical analysis. The structure of the assemblages and their relationships between individual sampling sites were clarified by shade plot and multivariate data analyses. The analysis of dominant species vitality at sampling sites and their autecology gave the foundation for modification of taxa data matrix and recalculation the diatom indices. Biological assessment showed that one of the artificial ponds constructed at the stream channel was characterized by good ecological status, and its presence strongly affected the state of the downstream ecosystem following the development of a unique assemblage of diatoms that prefer oligosaprobic and oligotrophic waters. The presence of these species was also noted in the downstream sections, but most of the cells were dead. As the indicator values of these taxa are high, their presence artificially increased the ecological status of the stream, resulting in the hydrochemical assessment not being in line with the bioassessment. Therefore, a new procedure was adopted in which non-characteristic taxa for the downstream sections were excluded from analysis. This approach corrected the results of bioassessment characterizing the ecological status of the stream as poor along its entire course, with the exception of this unique pond. For hydromorphologically transformed streams and rivers with disturbed channel continuity, the correct result of an incorrect diatom-based bioassessment may be retrieved after excluding species unusual for the type of ecosystem from the studied assemblages, i.e., the species which are unable to reproduce in that area and are only carried into it by the water flow. Assessment of the ecological status of aquatic ecosystems based on biotic factors is an essential tool of aquatic ecosystems monitoring in many countries. This type of assessment requires a multifaceted approach, in particular, to identify factors that may disrupt this assessment. Standardization of biomonitoring methods is an important step in correct assessment; thus, the findings of this paper will be useful in routine biomonitoring around the world.

## Introduction

Water ecosystems have always been an important city-forming factor (Arandjelovic & Bogunovich, 2014; Cieślak-Arkuszewska, 2020). Unfortunately, in urban areas, river beds are usually heavily modified; thus, they lost their natural course (Winiwarter et al., 2016; Mackin & Lewin, 2018). They are characterized by large hydromorphological transformations related to human activity, such as those based on the regulation and concreting of their beds, or by the introduction of artificial reservoirs along their course. Each urban river and stream ecosystem is a specific environment, and various biotic and abiotic elements may affect the results of bioassessment; therefore, each river and stream, especially those with highly-modified channels and with disrupted ecological continuum should undergo its own unique procedure.

According to the river continuum concept, the environmental conditions and communities of organisms in flowing water ecosystems gradually change along the course of a river (Vannote et al, 1980). Hydrobiological conditions shape specific communities of aquatic organisms, with these being characteristic, *i.e.,* natural or typical, of individual sections of ecosystem (Freedman et al., 2014; Stenger-Kovács et al., 2014; Backus-Freer & Pyron, 2015). However, this concept works if the river channel is of a natural type and has not been subjected to any hydromorphological reconstructions. In such ecosystems, the results of a bioassessment are not affected by any interference and reflect the true ecological status. The situation changes when the river undergoes hydromorphological transformations, such as river channel straightening, supporting the river banks with concrete slabs, closing the river into an underground channel or slowing down the river flow by the construction of artificial reservoirs. These transformations strongly modify the hydrological regime and the hydrochemical conditions, thus altering the aquatic communities into a form far from ‘natural’ for the type of environment *i.e.,* lotic ecosystems become full of lentic-type organisms. Therefore, any bioassessment of water quality and ecological status based on these modified communities may yield incorrect results, as demonstrated by Żelazna-Wieczorek & Nowicka-Krawczyk (2015); Szczepocka, Żelazna Wieczorek & Nowicka-Krawczyk (2019).

The assessment of the ecological status of surface waters in routine biomonitoring is based on four biotic factors: diatoms, aquatic invertebrates, fishes and macrophytes (European Union, 2000). As the development of benthic diatom assemblages strongly depends on hydromorphological and hydrogeochemical factors, diatom material can be recognised as a highly reliable biomarker reflecting most of the environmental conditions of the ecosystem (Descy, 1979; Hofmann, 1994; Van Dam, Mertens & Sinkeldam, 1994; Kelly & Whitton, 1995; Lavoie et al., 2014; Charles et al., 2021). Long-term studies on the autecology of diatoms have allowed species to be assigned ecological indicator values, these being specific numbers reflecting the preference range of a single species for particular environmental parameter. In diatom-based biomonitoring, indicator values are the foundation for calculating diatom indices, giving direct information about the state of the environment (Round, Crawford & Mann, 1990; Kelly et al., 2012). For over 20 years, diatom indices have been used worldwide to assess the state of flowing and standing water ecosystems (Kelly et al., 2008; Stevenson, 2014; Holmes & Taylor, 2015; Kahlert et al., 2016; Charles et al., 2021).

As diatoms are used as indicators for such a wide range of purposes, the bioassessment procedures may incur some errors that cannot be avoided. Disturbances in river/stream continuity usually result in incorrect results being obtained for the ecological status assessment, indicated by large differences being observed between individual sections of the ecosystem (Szczepocka, Żelazna Wieczorek & Nowicka-Krawczyk, 2019; Charles et al., 2021; Latinopoulos et al., 2021).

A good site for researching how hydromorphological transformations affect the procedure of bioassessment is the Łódź Municipal Agglomeration (Central Poland), where many streams were reconstructed in the 19th century as part of the development of the textile industry in the city. One of the streams located at the southern part of the city, the Olechówka, is highly transformed. Its channel contains regulated sections, most of which are in the open air and hidden underground at the spring section; in addition, the stream channel is frequently disrupted by small artificial reservoirs (ponds) where water mills used to operate. The stream has previously been used as an area for hydrobiological research on ichthyofauna (Kruk et al., 2017) and aquatic macroinvertebrates (Tszydel et al., 2015; Tszydel, Markowski & Majecki, 2016). Due to its location, at the outskirts of the city, the stream was characterized by a high diversity of fish species and macroinvertebrates compared to other streams in Łódź. In addition, the hydrochemical conditions of the stream were also investigated in 2015 –2018 to determine the state of the stream using chemical markers (Bagrowicz et al., 2017; Fortuniak et al., 2018; Ziułkiewicz et al., 2019).

A biological assessment is valid only when it is based on living organisms (Dolédec, Statzner & Bournard, 1999; Gillett, Oudsema & Steiman, 2017). Only living species can accurately determine the environmental conditions in aquatic ecosystems. Their ability to reproduce in the conditions prevailing in a given ecosystem allows them to be considered as good indicators of ecological status (Rimet & Bouchez, 2012; Charles et al., 2021).

Since biomonitoring is strongly based on the autecology of living diatoms, *i.e.,* by calculating diatom indices, hydromorphological transformations that change assemblages into those which are far from ‘natural’ typically results in incorrect ecological status assessments. This paper verifies the hypothesis that for hydromorphologically transformed streams and rivers with disturbed channel continuity, the correct result of an incorrect diatom-based bioassessment may be retrieved after excluding species unusual for the type of ecosystem from the studied assemblages, *i.e.,* the species which are unable to reproduce in that area and are only carried into it by the water flow.

The main purpose of the work was to identify the disturbances associated with biological assessment of the ecological status of stream resulting from impact of artificial ponds on of flowing waters (interruptions in channel continuity), and to propose a new procedure for the diatom-based biomonitoring of this type of ecosystems. The new procedure is based on a detailed investigation of diatom assemblage structure, one that takes into account its qualitative structure, ecological preferences of species and changes in the quantities of species along the watercourse.

## Material and methods

### Study area

The research area includes the Olechówka, a stream located in the Łódź Municipal Agglomeration (Central Poland, Europe). The city of Łódź is characterized by the presence of small, fast-flowing streams, which played a key role in the development of its textile industry at the beginning of the 19th century. The increasing amounts of sewage produced by the rapidly developing agglomeration were drained directly into streams through gutters and ditches, especially in the city centre (Kruk et al., 2017). The Olechówka is a 12.5 km long watercourse located on the outskirts, in the southern part of the city. Due to its location, the stream was less polluted than those crossing the central part of the city. In the mid-nineteenth century, numerous ponds were created as places of recreation for the surrounding population and several water mills operated on the stream course.

**Figure 1:**
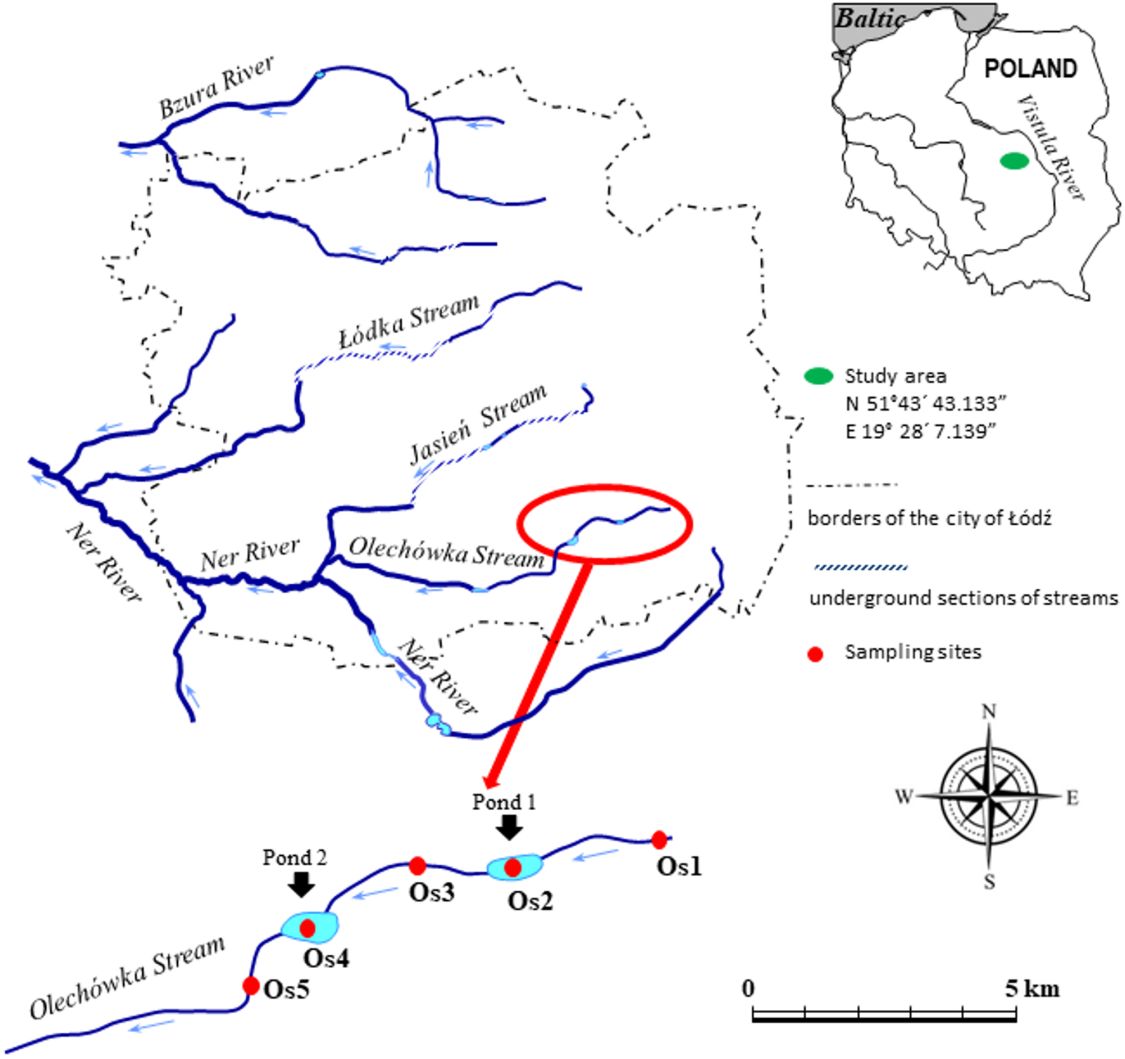
Study area with the location of sampling sites. The code of sampling site consists: letter of the stream (O –the Olechówka stream), and digit expressing the site number preceded with “s”. Pond 1 (Os2)–Olechowska pond, pond 2 (Os4)–Tomaszowska pond.

The research was conducted at the upper section of the Olechówka. Five sampling sites were selected along the length of the stream (Fig. 1). Site 1 was located at the spring section of the stream on the edge of the Municipal Park of the Olechówka Spring; site 2 was located approximately 800 m below site 1, at the basin of the first pond (Olechowska pond); site 3 was chosen at the stream course ca 400 m below Olechowska pond; site 4 was located at the second pond (Tomaszowska pond); and site 5 at a location around 400 m below Tomaszowska pond. The Olechówka stream emerges from an underground channel next to the outlet of a rain/sewage collector from the Olechów residential district. It is located a few hundred meters from another sewage collector draining sewage from the railways and the intermodal transport industry area. The presence of both sewage collectors affects the ecological status of the stream directly from its sources.

The hydrochemical background of the stream was assessed during the present hydrobiological surveys similar to previous studies (Szczepocka, Żelazna Wieczorek & Nowicka-Krawczyk, 2019). The ranges and mean values of the parameters are given in Table S1.

### Analysis of diatom assemblages

Phytobenthic samples of diatoms were collected in October 2017, January, March, May, July and August 2018 from zones of mid-speed water current. Diatom material was collected according to the procedure described in Szczepocka, Żelazna Wieczorek & Nowicka-Krawczyk (2019). In total, 23 fresh samples were collected. Each sample was coded according to the following scheme: a stream name code (O –the Olechówka), followed by two digits of the year of sampling, two digits of the month of sampling, and the ‘S’ symbol followed by the number of the sampling site. The samples were initially observed while fresh to assess whether the diatoms were alive or not. Fresh diatom samples from all study sites were investigated in the Nikon Eclipse 50i light microscope with 10 × 40, 10 × 60 magnification in order to evaluate the percentage of living/dead cells. The correct ecological state assessment should be based on taxa occurring with 90% in living forms (Zgrundo Peszek & Poradowska, 2018). Moreover, in the case of taxa which occurred in 90% of dead forms at river sites below the reservoirs and simultaneously they dominated in reservoirs in living forms, they were excluded from bioassesment.

The qualitative analysis was performed according to Hofmann, Werum & Lange-Bertalot (2011) and Lange-Bertalot et al. (2017) while the quantitative analysis Cholnoky (1968). The dominant species were distinguished according to Rakowska (2001). Since dominant species have the most significant impact on the results of biological assessment, for the purpose of the study, this group was additionally divided into three subgroups depending on a percentage share in assemblages (^%^SH): species of low dominance (low-dominant, LD) with 5% ≥ ^%^SH >11%, species with medium dominance (medium-dominant, MD) with 11% ≥ ^%^SH >21%, and species with high dominance (high-dominant, HD) ^%^SH ≥ 21%. The species constancy was determined according to the Braun-Blanquet scale (Bohr, 1962).

The samples were grouped based on their qualitative and quantitative similarities in diatom assemblage structure. The initial step in the analysis was performed to reduce the species set and adjust the matrix to a specified number of species (*n* = 50, transformed by square root) by including only the species important for the next comparison of the studied samples. To align the plots with regard to the range and quality of data, a Shade plot was used. The plot displays a matrix with species (rectangles gray-shading shows the abundance of diatom species, scale was expressed as Log (X+1); white indicated an absence of species while black indicated maximum abundance). The samples were grouped by hierarchical clustering analysis (HCA) using Bray-Curtis similarity coefficient on transformed data (Clarke et al., 2014).

To summarize the variation in species composition and quantity and to interpret this summary with the help of the best-fitting subset of hydrochemical parameters, the multivariate data analyses were performed. The response data were compositional with 2.2 SD gradient; therefore, the Constrained linear method—a Principal Component Analysis with supplementary variables was chosen. For the response data centering by species was applied without any data transformation. The analysis was preceded by Interactive-forward-selection test to select the best-fitting hydrochemical factors for interpreting the summary in species composition. Factors with significance level *P* ≤ 0.05 were chosen for PCA analysis. Multivariate data analyses were performed using Canoco for Windows 5.0 Software.

### Ecological status assessment

The ecological status of the Olechówka stream was assessed based on the following diatom indices: IPS –Specific Pollution Sensitivity Index (CEMAGREF, 1982), GDI –Generic Diatom Index (Coste & Ayphassorho, 1991), IBD –Biological Diatom Index (Lenoir & Coste, 1996), and TDI –Trophic Diatom Index (Kelly & Whitton, 1995). The IPS, GDI and TDI indices were calculated using OMNIDIA 6.0 software (Bordeaux, France). The ranges of the diatom indices, their respective ecological status and trophic status were adapted according to Dumnicka et al. (2006) to obtain the IPS, GDI, IBD and TDI indices (see: Szczepocka, Nowicka-Krawczyk & Kruk, 2018, Fig. 2). The diatom autecology was checked in OMNIDIA 6.0 software database. In order to verify the research hypothesis, the ecological status assessment was subjected to a second modelling procedure using OMNIDIA software; this time, diatom taxa considered ‘atypical’ for a particular section of the stream were eliminated from the analysis. Taxa were designated as ‘atypical’, if their presence in the section of a stream was not in line with their ecological preferences, and simultaneously its participation in assemblages was lower than that observed in the upper section as a result of inhibited reproduction. As the previous studies revealed, in hydromorphogically transformed rivers the presence of the *Achanthidium minutissimum* s.l. falsify the results of bioassessment of the ecological status; therefore this species was also removed from data matrix (Szczepocka, Żelazna Wieczorek & Nowicka-Krawczyk, 2019).

**Figure 2:**
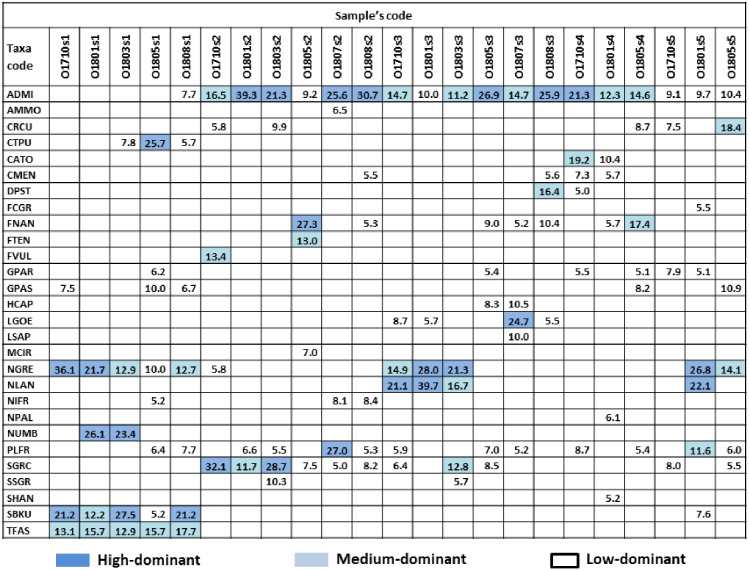
Dominant species and their percentage in samples. Colour indicate dominant subgroups: HD (high-dominat) ^%^SH ≥ 21%, dark blue; MD (medium-dominat) 11% ≥ ^%^SH >21%, light blue; LD (low-dominat) 5% ≥ ^%^SH >11%, white. Diatom species code: ADMI–*Achnanthidium minutissimum* ss, AMMO–*Halamphora montana*, CRCU–*Craticula cuspidata*, CTPU–*Ctenophora pulchella*, CATO–*Cyclotella atomus*, CMEN–*C. meneghiniana*, DPST – *Discostella pseudostelligera*, FCGR–*Fragilaria gracilis*, FNAN–*F. nanana*, FTEN–*F. tenera*, FVUL–*Frustulia vulgaris*, GPAR–*Gomphonema parvulum*, GPAS–*G. saprophilum*, HCAP–*Hippodonta capitata*, LGOE–*Luticola goeppertiana*, LSAP–*L. saprophilum*, MCIR–*Meridion circulare*, NGRE–*Navicula gregaria*, NLAN–*N. lanceolata*, NIFR–*Nitzschia frustulum*, NPAL–*N. palea*, NUMB–*N. umbonata*, PLFR–*Planothidium frequentissimum*, SGRC – _*Stauroneis gracilis*_, SSGR–*S. subgracilis*, SHAN–*Stephanodiscus hantzschii*, SBKU–*Surirella brebissonii* var. *kuetzingii*, TFAS–*Tabularia fasciculata*.

To summarize the similarity in species composition between samples in relation to diatom indices, a non-metric multidimensional scaling (NMDS) with an additional principal component analysis (PCA) rotation step of NMDS was used. Two analyses were performed in parallel: the first included all diatom taxa occurring in samples (pre-modelling analysis), while the second (post-modelling analysis) used revised diatom indices, which were calculated after the species discarded from the modelling procedure of ecological status were excluded from the data matrix. In both cases, analyses were based on a Bray-Curtis similarity coefficient with a solution on four axes. Special attention was paid to the changes occurring along the stream course in Spring and Autumn, these being the most crucial seasons for diatom-based biomonitoring (Szczepocka & Żelazna Wieczorek, 2018). Since previous studies have indicated IPS to be the most reliable diatom index (Szczepocka, Nowicka-Krawczyk & Kruk, 2018), the attribute plots in the XY ordination space of IPS and PCA rotation of NMDS were created for both study seasons.

## Results

### Hydrochemical background of the stream

The water of the Olechówka stream was circumneutral or slightly alkaline - mean pH varied between 7.1 and 7.3 (Table S1). The electrolytic conductivity varied between sampling sites. The highest value was recorded at the spring section of the stream (site 1), the mean value was 888 µScm^−1^, while the lowest was observed at the first pond (site 2 - Olechowska pond) with 413 µScm^−1^ (Table S1). The highest concentration of dissolved oxygen - mean value up to 9.4 mgl^−1^ was recorded at site 1; similar values were observed at other sites, ranging from 3.3 mgl^−1^ (site 2) to 4.6 mgl^−1^ (site 5). The mean biological oxygen demand was the lowest at the spring section, *i.e.,* site 1 (2.9 mgl^−1^), and the highest at site 2 (9.7 mgl^−1^); the value decreased downstream (Table S1). The highest mean total nitrogen was observed at site 1 (4.09 mgl^−1^), this value was close to 2.00 mgl^−1^ at the remaining sites. Both the lowest concentration of N-NH_4_ and the highest of N-NO_3_ were recorded at site 1 (mean values of 0.043 mgl^−1^ and 2.83 mgl^−1^, respectively), and the highest concentration of N-NH_4_ was recorded at site 2, *i.e.,* Olechowska pond, with a mean value of 1.6 mgl^−1^. In addition, a high mean concentration of N-NO_3_ was also recorded at site 4, Tomaszowska pond, (2.5 mgl^−1^), while values around 0.6 mgl^−1^ were observed for other study sites. Similar mean total phosphorus levels were found at all study sites, ranging from 0.12 mgl^−1^ to 0.16 mgl^−1^. Sites 1 to 3 in the upper section of the stream were found to have similar mean P-PO_4_ concentrations, *i.e.,* between 0.080 and 0.085 mgl^−1^; however, it exceeded 0.13 mgl^−1^ at the second pond (site 4) and increased up to 0.2 mgl^−1^ in the lowest section of the stream (site 5) (Table S1).

### Analysis of diatom assemblages

In total, 139 diatom taxa were identified in 23 phytobenthic samples collected from the Olechówka stream and among these, 28 taxa were recognized as dominants. The following dominant and constant species were present in all samples and alive in fresh material with >95% participation at all sites: *Achnanthidium minutissimum* s.s. Kützing (present in 100% samples as HD in 7/23 samples), *Planothidium frequentissimum* (Lange-Bertalot) Lange-Bertalot (present in 100% samples, as LD in 11/23), *Gomphonema parvulum* (Kützing) Kützing (present in 100% samples, as LD in 6/23) and *Navicula gregaria* Donkin (present in 91% samples, as HD in 5/21). In addition, *Gomphonema saprophilum* (Lange-Bertalot & E. Reichardt) Abraca, R. Jahn, J. Zimmermann & Enke and *Nitzschia frustulum* (Kützing) Grunow, were present in 83% of samples, but the threshold percentage value for dominant species was exceeded only in a few samples (Fig. 2).

The analysis also revealed a group of taxa which dominated at the spring section of the stream (site 1) and gradually disappeared along the stream course –*Ctenophora pulchella* (Ralfs ex Kützing) Williams et Round, *Nitzschia umbonata* (Ehrenberg) Lange-Bertalot, *Surirella brebissoni* var. *kuetzingii* Krammer et Lange-Bertalot, and *Tabularia fasciculata* (C. Agardh) Williams et Round. Moreover, some taxa were characterized by high dominance in a particular section, but one that slightly decreased downstream: *Fragilaria nanana* Lange-Bertalot, *F. tenera* (W. Smith) Lange-Bertalot, *Stauroneis gracilis* Ehrenberg, and *S. subgracilis* Lange-Bertalot et Krammer (Fig. 2). The percentage of living cells of the above species in fresh samples collected at site 2 was above 90%.

In fresh samples collected downstream, species such as *Fragilaria nanana, F. tenera, Stauroneis gracilis, S. subgracilis* that develop in large quantities at site 2 (Olechowska pond) were mostly dead. The percentage of living cells in fresh material was below 10% and it was decreasing downstream.

**Figure 3:**
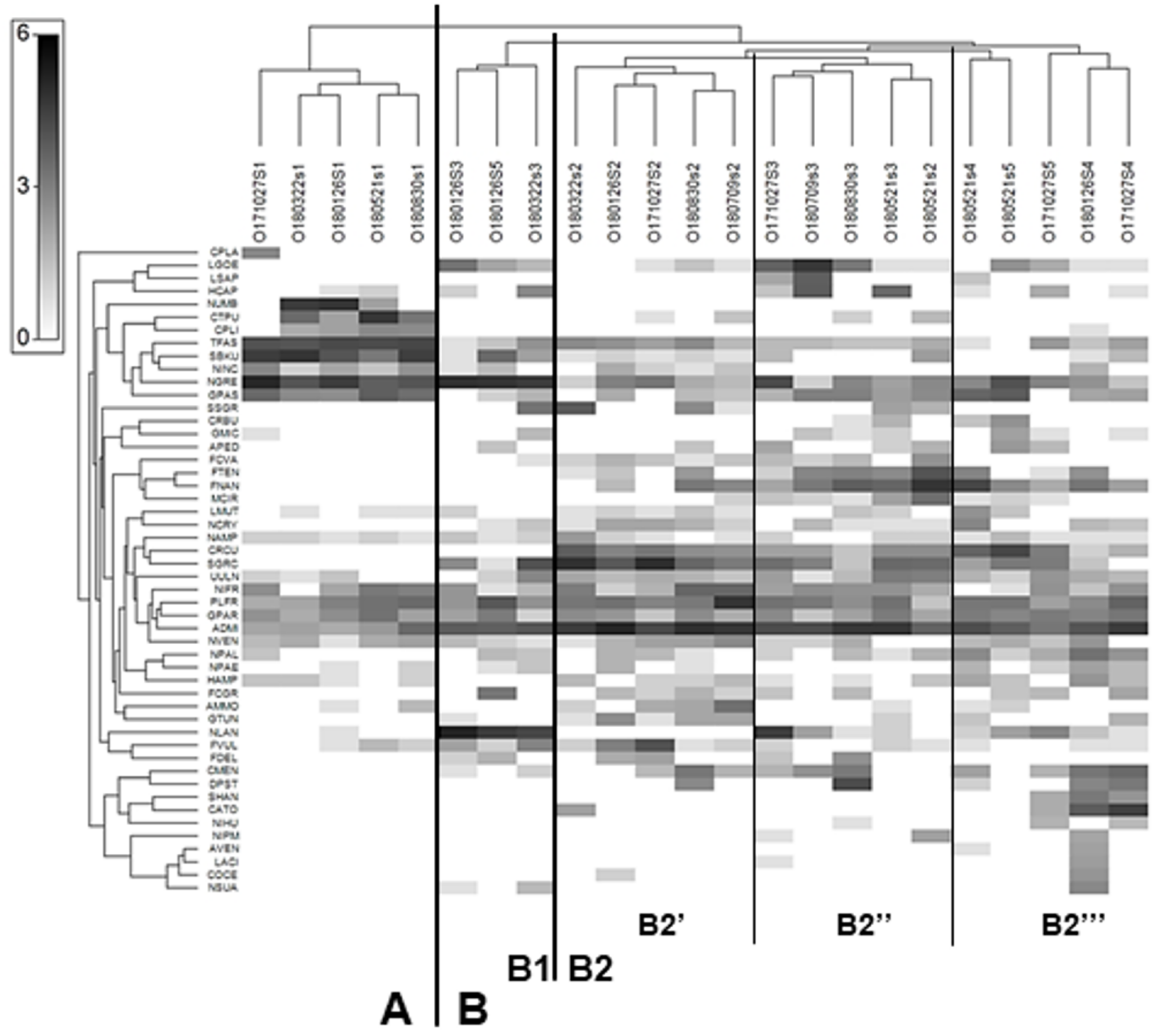
Shade plot of abundance of 50 most important diatom species in assemblages from the Olechówka stream. Grey-scale intensity (abundance scale expressed as Log (X+1)). Samples were grouped using Bray-Curtis similarities on the transformed data, by HCA. Diatom species code: ADMI - *Achnanthidium minutissimum* ss, AMMO–*Halamphora montana*, APED–*A. pediculus*, AVEN–*Halamphora veneta*, CRBU - *Craticula buderi*, CRCU–*C. cuspidata*, CTPU–*Ctenophora pulchella*, CATO - *Cyclotella atomus*, CMEN–*C. meneghiniana*, COCE–*C. ocellata*, CPLA - *Cocconeis placentula,* CPLI–*C. lineata*, DPST - *Discostella pseudostelligera*, FCGR - *Fragilaria gracilis*, FCVA–*F. vaucheriae*, FDEL–*F. delicatissima*, FNAN–*F. nanana*, FTEN–*F. tenera*, FVUL–*Frustulia vulgaris*, GMIC–*Gomphonema micrpus*, GPAR–*G. parvulum*, GPAS–*G. saprophilum*, GTUN –*G. truncatum*, HAMP–*Hantzschia amphioxys*, HCAP –*Hippodonta capitata*, LACI–*Luticola acidoclinata*, LGOE–*L. goeppertiana*, LMUT–*L. mutica*, LSAP–*L. saprophilum*, MCIR - *Meridion circulare*, NCRY–*Navicula cryptocephala*, NGRE–*N. gregaria*, NLAN - *N. lanceolata*, NVEN–*N. veneta*, NAMP - *Nitzschia amphibia,* NIFR–*N. frustulum*, NINC - *N. inconspicua*, NIHU–*Trybionella hungarica*, NPAL–*N. palea*, NPAE–*N. paleacea,* NIPM - *N. perminuta*, NSUA–*N. subacicularis*, NUMB–*N. umbonata*, PLFR–*Planothidium frequentissimum*, SGRC -*Stauroneis gracilis*, SSGR - *S. subgracilis*, SHAN–*Stephanodiscus hantzschii*, SBKU–*Surirella brebissonii* var. *kuetzingii*, TFAS–*Tabularia fasciculata,* UULN –*Ulnaria ulna*.

The Shade plot grouped all samples into two main clusters –A and B. Cluster A comprised all samples from site 1, while cluster B included the samples from all sites. The analysis divided cluster B into subclusters B1 and B2, and further divided the latter into additional B2’, B2” and B2”’ subgroups (Fig. 3). As a result of Shade plot transformation, 50 species were identified as type-characteristic, *i.e.,* highly influencing the structure of diatom assemblages within each of the clusters, subclusters and groups. Cluster A was unique; it could be distinguished from the others as a result of its high participation of *Nitzschia umbonata, Ctenophora pulchella, Surirella brebissoni* var. *kuetzingii, Tabularia fasciculata, Navicula gregaria* and *Gomphonema saprophilum*. All these taxa, with the exception of *N. gregaria*, reached high dominance only at site 1; they did not appear downstream or their share significantly decreased.

Subcluster B1 included samples from site 3 collected in January and March and a sample from site 5 collected in January. The species that dominated at this subcluster were *Navicula lanceolata* and *N. gregaria*. The first subgroup of subcluster B2 (B2′) contained samples only from site 2 (Olechowska pond); the dominant species characteristic of this site were *Fragilaria nanana, F. tenera, Stauroneis gracilis, S. subgracilis, Craticula cuspidata, Achnanthidium minutissimum* s.s. The presence of these taxa also affected sites below Olechowska pond. Subgroup B2” included samples from site 3 collected in May, July, August and October and a sample from site 2 collected in May. Some species characteristic of the previous group were also found to demonstrate large shares in this group, while *Luticola goeppertiana, L. saprophila, Hippodonta capitata, Navicula lanceolata* and *Discostella pseudostelligera* were also of high importance for B2”.

The last group, B2”’, included all samples from site 4 (Tomaszowska pond) and two samples from the last section of the stream, *i.e.,* those collected in May and October from site 5. B2”’ was also shaped by taxa from the two previous groups, as well as centric diatoms characteristic of lentic ecosystems: *Cyclotella atomus, C. meneghiniana, Discostella pseudostelligera* and *Stephanodiscus hantzschii*. The dominant taxon that differentiated all samples from cluster B, but was also present at much lower proportions in samples of cluster A, was *Achnanthidium minutissimum* s.s.

Hydrochemical factors (Table S1) explained 54.4% of the total variation in diatom composition in samples. Interactive-forward-selection test indicated that the highest influence on the variation of taxa in assemblages had the total nitrogen (Total N) –it contributed 30.5% at the total variation with the significance level *p* < 0.001. Other statistically significant factors *p* ≤ 0.005 were: EC –21.8%; DOC –19.4%; and N-NO_3_ –18.6% of contribution in the total variation of diatom distribution.

**Figure 4:**
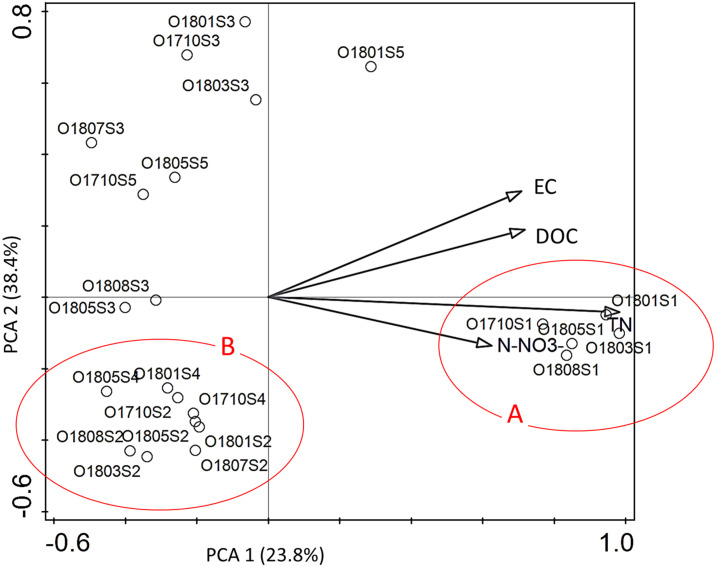
The PCA ordination plot displaying the relations between the samples of diatoms collected from the Olechówka stream and environmental variables. Environmental variables most significant p ≤ 0. 005 for shaping diatom assemblages at the stream; (A) samples from the source section of the stream–site 1; (B) samples from ponds located at the stream course: site 2, Olechowska Pond; site 4, Tomaszowska Pond.

All statistically significant principal components showed positive mutual correlation to diatom assemblages at the site 1 (Fig. 4). All diatom samples from ponds located at the stream (sites 2 and 4) were grouped together in III ^rd^ quarter of ordination space with negative correlation to EC and DOC. The rest samples from river sections located below the reservoirs were grouped in a distance to each other without any regularity.

### Ecological status assessment

**Figure 5:**
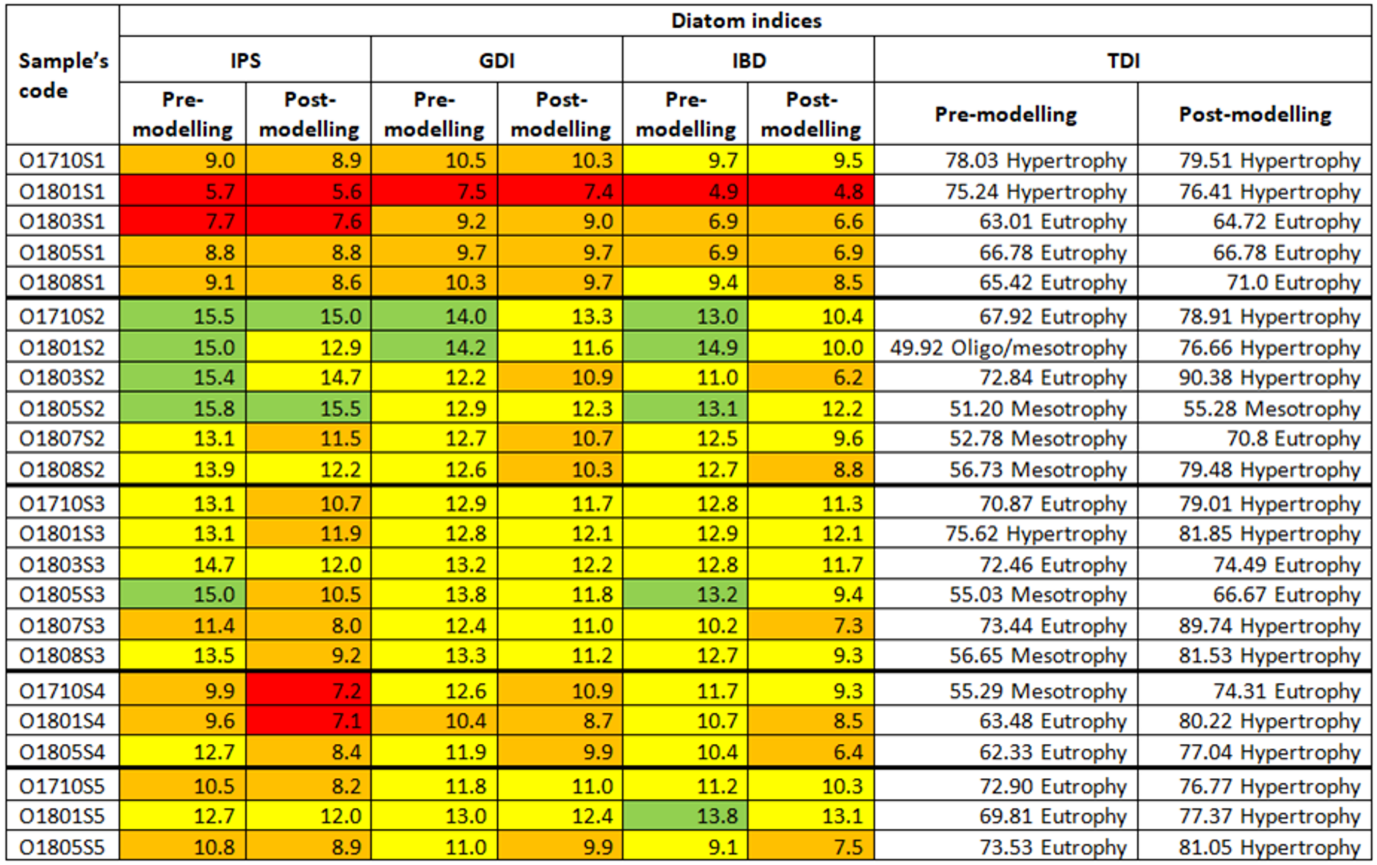
Changes in the ecological status of samples from the Olechówka stream assessed based on pre-modelling and post-modelling bioassessment procedure.

The ecological status of the Olechówka sites ranged from good to bad: the assessment was based on three diatom indices, *i.e.,* IPS, GDI and IBD, calculated with the use of an unmodified data matrix (Fig. 5). The lowest statuses (bad, poor) were recorded in the spring section of the stream (site 1), while the highest (good, moderate) were noted at the first pond: Olechowska pond (site 2). The ecological status below Olechowska pond remained at a moderate level. Site 3, affected by the waters flowing down from Olechowska pond, was characterized by the best water quality of all sections of the stream; however, samples O1710S4, O1801S4, O1710S5 and O1805S5 taken a little further downstream indicated poor water quality. Assessment based on TDI index indicated eutrophic status for most samples. The highest trophic level was noted at the spring section of the Olechówka stream (site 1), and the lowest at the first pond: Olechowska pond (site 2) (Fig. 5).

The ecological status recorded for the Olechówka stream indicated that the water quality of site 2, Olechowska pond, had a significant impact on the quality of sites located downstream. Diatom species developing in high numbers at site 2 affected the bioassessment of sites along the course of the stream. The quantitative and qualitative analysis of dominant species from site 2 and downstream sections showed that dominants can be divided into two groups. The first group contained species occurring with a similar number of valves at all mentioned sites; the cells of these species from fresh material were alive, and hence were growing and reproducing in assemblages at the sections of the stream below Olechowska pond: *Achnanthidium minutissimum* s.s.*, Craticula cuspidata, Navicula gregaria, N. lanceolata* and *Planothidium frequentissimum*. The second group comprised dominants whose number decreased downstream of Olechowska pond: *Fragilaria nanana, F. tenera, Stauroneis gracilis* and *S. subgracilis*; moreover, while investigating fresh material, in this case 90% of cells were found to be dead.

As most of the cells in this second group were already found to be dead in fresh material, *i.e.,* they were not able to grow or reproduce downstream of the pond, a second assessment of ecological status was carried out based on a revised data matrix that excluded these species from the second group, together with *Achnanthidium minutissimum* s.s., as proposed by Szczepocka, Żelazna Wieczorek & Nowicka-Krawczyk (2019). As a result, that IPS, GDI, IBD and TDI indices were recalculated using OMNIDIA software. The recalculated values indicated that the IPS, GDI, IBD indices decreased in all study samples, while TDI increased (Fig. 4). The spring section of the Olechówka stream (site 1) was now characterized by poor to bad ecological status. The first pond, Olechowska pond, maintained the best ecological status (good to moderate); however, the sites below the pond now demonstrated a lower status, even by a whole level in the case of IPS index (Fig. 5).

**Figure 6:**
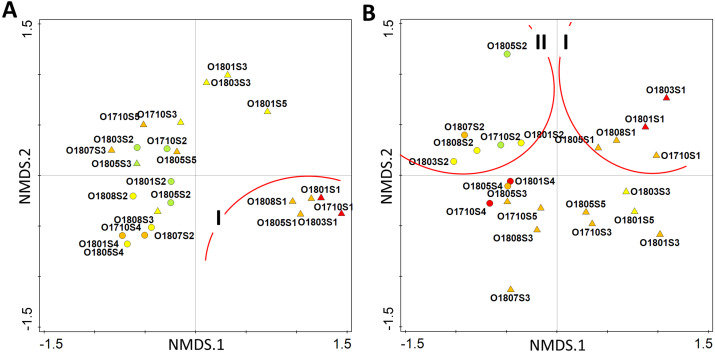
Distribution of samples according to the similarity of diatom assemblages with diatom indices as supplementary variables by pre-modelling analysis (A) and post-modelling analysis (B). Circles–pond sites, triangles–stream sites, colour indicate the ecological status–red bad status, orange–poor status, yellow–moderate status, green–good status.

The multivariate analysis of all identified taxa suggested that the stream has a poor ecological status only in the upper section (site 1), while in its further course (site 2 and 3) it achieves good ecological status. The percentage of good-moderate status samples was 56.5% along the whole stream. The analysis placed samples collected from site 1 in the 4th quarter of ordination space (group I), while rest of samples were grouped together between the 2nd and 3rd quarters (Fig. 6A). The modification of the data matrix performed for the recalculation of diatom indices clearly changed the distribution of samples in two-dimensional ordination space. The samples from the upper section of the stream were now placed in the 1st quarter of the ordination space (group I), while all those from the second site, *i.e.,* Olechowska pond, could be found in the 2nd quarter (group II) (Fig. 6B). The remaining samples from the stream sections, together with those from Tomaszowska pond, were placed below the horizontal axis, across the 3rd and 4th quarters of the ordination space (Fig. 6B). Only two samples from Olechowska pond collected in April and October had good ecological status, and the share of their good-moderate samples was 34.8%.

**Figure 7:**
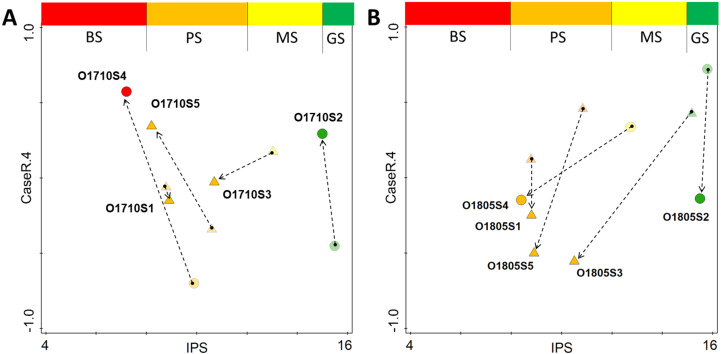
Changes in distribution of samples across attribute plot based on IPS diatom index and PCA rotation of NMDS on 4th axis for Autumn (A) and Spring (B) season. Single arrow point out the change in sample location from pre-modelling to the post-modelling analysis; BS (red) bad ecological status; PS (orange) poor ecological status; MS (yellow) moderate ecological status; GS (green) good ecological status; circles–pond sites; triangles–stream sites.

Regarding the changes observed between the pre- and post-modelling analyses with regard to sample distribution along the stream course, the lowest change in both ecological status and spatial distribution in the attribute plot, was recorded for site 1 of the stream (Figs. 7A–7B). The inclusion of the revised data into the analysis changed the spatial distribution of samples across the plot, and the greatest change was recorded for samples collected below Olechowska pond (sites 3, 4 and 5). In addition, the post-modelling analysis demonstrated a lower distribution of samples along the NMDS gradient for the pre-modelling analysis: for both seasons, with the spread of samples along the 4th axis of NMDS decreasing by 0.2 pts after recalculation. The revised analysis characterized Olechówka stream as having poor to bad ecological status; however, a good status was maintained in Olechowska pond.

## Discussion

The Olechówka stream is excellent example of watercourses with a disturbed channel continuum model. Most of the Olechówka streambed is hydromorphologically transformed—the artificial ponds have been created at its watercourse. Like other streams in Łódź city, the Olechówka has poor water quality. The electrolytic conductivity even within the spring section is very high, reaching 1115 µScm^−1^. Its physical and chemical parameters, *i.e.,* dissolved oxygen, pH, DOC_5_, total nitrogen, ammonium, nitrate, total phosphorus and phosphate content, confirms that from its origin, the stream is heavily polluted (Table S1). The poor water condition of the stream has also been confirmed by other hydrobiological and physical and chemical analyses (Bagrowicz et al., 2017; Fortuniak et al., 2018; Ziułkiewicz et al., 2019). Previous research using diatom indices also found both the spring section and the downstream range to be in poor ecological condition (Szczepocka, Nowicka-Krawczyk & Kruk, 2018). The present assessment based on IPS, GDI, IBD, TDI indices indicated bad to poor ecological status and an eutrophic state at the spring section, but the ecological status of the sites located below the first of the artificial ponds: Olechowska pond (site 2) increase significantly, even by two levels to good/moderate status (Fig. 5). Differences were also revealed between site 1 and other sampling sites by the Shade plot analysis (Fig. 3). All samples from site 1 were included in a separate cluster, *i.e.,* cluster A, while the remaining ones were gathered in a significantly different cluster, *i.e.,* cluster B. The high ecological status level (good, moderate) of site 2 directly increased those of sites located below the pond; this was a surprising phenomenon, as the assemblages downstream from the pond were dominated by taxa whose autecology indicates highly polluted waters with high eutrophic status: *Luticola goeppertiana, L. saprophila, Navicula gregaria,* and *Nitzschia frustulum* (Lange-Bertalot et al., 2017). Therefore, the site of Olechowska pond was definitely responsible for the increase in the level of ecological status. The Olechowska pond (site 2) had a distinctly different species composition, which affected the assemblages structure along the stream course (at sites 3, 4 and 5) by the runoff of species from the pond to the downstream sections. All samples beginning from Olechowska pond were grouped in cluster B by the Shade plot analysis, and the assemblages from this artificial pond appeared to have a clear influence in the creation of subcluster B2. The structure of diatom assemblages from Olechowska pond was shaped by the presence of dominants, such as *Fragilaria nanana, F. tenera, Stauroneis gracilis, S. subgracilis*, which are recorded in the OMNIDIA database and according to Lange-Bertalot et al. (2017) as being indicators of good quality, oligosaprobic and oligomesotrophic waters. The presence of the above dominants indicated good ecological status at Olechowska pond (site 2); however, this was not surprising, because the hydrochemical conditions were also characteristic of sites with high ecological status, *i.e.,* EC values were lower at Olechowska pond than in other sections of the stream, by even 197 µScm^−1^. The good status of water at Olechowska pond is result of a low depth of groundwater aquifer (up to 1 m). Oligotrophic and oligosaprobic water from the aquifer is mixing with the stream water at the pond creating unique environmental conditions. The centric diatoms such as: *Cyclotella* sp*. Discostella* sp*., Stephanodiscus* sp. as being characteristic for lentic ecosystems, were not recorded in Olechowska pond (Houk, Klee & Tanaka, 2010; 2014).

Analysis of the structure of diatom assemblages showed that dominant species of the Olechowska pond were also found in large numbers below the reservoir, but their cells were dead. The presence of the species downstream was due to the cells runoff from the pond. In downstream sections environmental conditions were inadequate for these species development; therefore, most of the cells were dead. The runoff of algae often move the species downstream into different/non-optimal habitat conditions where they cannot develop in and begin to die (Leukart & Mollenhauer, 1997). Ninety percent of: *Fragilaria nanana, F. tenera, Stauroneis gracilis, S. subgracilis* cells were dead, what confirms that their presence at the site 3 was solely due to runoff from the reservoir.

The assessment of the ecological status should be carried out on the basis of the living diatom cells. Ideally, the ratio of living to dead cells should be 90% to 10%, respectively (Zgrundo Peszek & Poradowska, 2018). The assessment of the viability of the diatom material is crucial, because only on the basis of living diatoms can the current condition of the aquatic ecosystem be assessed (Gillett, Oudsema & Steiman, 2017). Some studies have been conducted to assess whether the percentage of living to dead diatom cells in the community can reflect environmental conditions or can be used as a measure of anthropopressure in streams and rivers (Stevenson, 2014; Gillett et al., 2011). The obtained results confirm the significant role of living diatoms in biological assessment; therefore, diatom indices should be calculated mainly on the basis of the living cells.

The diatom-based procedure was performed a second time with a revised model to confirm whether a more reliable picture of the ecological status, which better reflects the hydrochemical assessment, can be obtained by excluding the species which dominate at site 2, but do not reproduce in sections below the pond. The new procedure included the removal of site 2 dominants, whose presence resulted only from transfer with water flow, from sections below the pond: *Fragilaria nanana, F. tenera, Stauroneis gracilis* and *S. subgracilis*. The results indicated a decrease in the IPS, GDI and IBD indices, together with an increase of TDI, suggesting that the Olechówka stream is characterized by poor ecological status from the very beginning to the end, with the exception of Olechowska pond. Therefore, the impact force of the pond which initially artificially overestimated the ecological status of the stream, decreased. The results of the biological assessment made with the modified procedure were more reliable and better reflected the hydrochemical conditions of the stream. This was also confirmed by the multivariate analysis and a detailed investigation of sites along the stream in Spring and Autumn (Figs. 6–7). The Shade plot of the samples created before the modelling indicated a high degree of separation between assemblages at the first site; however, the revised modelling procedure, excluding samples from site 2, moved the results into a separate quarter of the ordination space. The revised procedure excluded five taxa out of a total of 139. The use of the new procedure shifted samples collected in Spring and Autumn from the stream sections: these were gathered together based on IPS index and assemblages, and separated from the site 2 samples (Fig. 7). The revised findings unified the ecological status of the stream but did not change the uniqueness and separateness of the first artificial reservoir –Olechowska pond.

Another dominant species was *Achnanthidium minutissimum* s.s., a species also recognised as being typical of waters of very good quality. It should be emphasized that this taxon is of questionable value in the context of biological assessment of water quality, as its taxonomic identification is subject to many errors. This is a small species comprising many varieties distinguished by ecological preferences, *e.g.*, *Achnanthidium minutissimum* var. *jackii*, *A. minutissimum* var. *saprophilum*, *A. minutissimum* var. *inconspicuum*, and they are difficult to recognise with the use of light microscope: it is therefore common for misidentifications to occur, or for all varieties to be recognized as *A. minutisssimum* species complex, resulting in the ecological state of the aquatic ecosystems being overestimated (Szczepocka, Żelazna Wieczorek & Nowicka-Krawczyk, 2019). Taking into consideration the above and since *Achnanthidium minutissimum* s.s. overestimates the results of biological assessment, this species was excluded also from the entire data matrix in the second assessment.

The assemblages of diatoms from the second of the studied ponds –Tomaszowska pond (site 4), were dominated by centric diatoms typical for stagnant water ecosystems as: *Cyclotella atomus, C. meneghiniana, Discostella pseudostelligera, Stephanodiscus hantzschii* (Lange-Bertalot et al., 2017). Cells of these dominants were reproducing intensively, they were alive at fresh material, as the environmental conditions of the pond were in accordance with their autecology (Houk, Klee & Tanaka, 2010; 2014). The presence of two species recorded previously at Olechowska pond (site 2) was also noted here: *Fragilaria nanana, Stauroneis gracilis*; however, the cells of these species were dead in fresh samples. As Leukart & Mollenhauer (1997) claim such presence may be a result of the stream runoff (Leukart & Mollenhauer, 1997). *Achnanthidium minutissimum* s.s. was dominant species at all sites of the stream and its cells were alive in 90% of each fresh material slide.

As the obtained results show, skipping a step of checking the viability of diatom cells may cause errors in the assessment of the ecological state of aquatic ecosystems, especially those with disturbed continuum. The analysis of cells viability in fresh diatoms material in the assessment of aquatic ecosystems should be the first step in the biological assessment procedure.

## Conclusions

In conclusion, our findings indicate that the results of biological assessment in flowing waters may be strongly affected by hydromorphological transformations, in this case, the presence of ponds, resulting in an interrupted continuum. The diatom assemblages developing in flow-through reservoirs have an impact on the communities from the downstream sections of the rivers and streams disturbing the lotic character of communities. Our findings indicate that the species dominating in the artificial ponds are carried by the water flow; however, since the downstream ecosystems do not favour their development and reproduction, they die but the valves are still present in smaller quantities in the phytobenthos. In the case of rivers and streams transformed by human impact, where such alteration involves the reconstruction of the channel, the structure of diatom assemblages should be carefully analysed with regard to their species composition, their quantity along the water course and their species autecology. If the diatom assemblages below the ‘transformation-point’, in this case the artificial ponds, demonstrate completely different species compositions to those upstream, and the dominant taxa from the ‘transformation-point’ are mostly dead, they should be removed from the biological assessment of the downstream sections.

The procedure of diatom-based bioassessment of rivers flowing through reservoirs should take into account the analysis of diatom cells in fresh, just collected samples. The first stage should include calculation of the percentage of living to dead diatom cells in unfixed material for all collected samples. The correct ecological state assessment should be based on taxa occurring with 90% in living forms. In addition, for river sites located below the reservoirs where is a high probability that diatoms cells flowing from the reservoir may occur, the comparative analysis of diatom assemblages in reservoir and below river site should be made. If some taxa occur in living forms with high percentage in the reservoir and concurrent most of these cells are dead in the river site below the reservoir, they should be excluded from biological analysis.

## Supplemental Information

10.7717/peerj.12457/supp-1 Supplemental Information 1 Raw data

10.7717/peerj.12457/supp-2 Supplemental Information 2 Range and average of the physical and chemical parameters of water at the sampling sites of the Olechówka stream

## Additional Information and Declarations

### Data Availability

The following information was supplied regarding data availability: The data are available in the Supplemental File.

## References

[ref-1] PN-EN ISO 5667-6: 2016-12. Jakość wody. Pobieranie próbek. Część 6: Wytyczne dotyczące pobierania próbek z rzek i strumieni (Water quality. Sampling. Part 6: guidelines for sampling rivers and streams). PNK Warszawa Poland (in Polish).

[ref-2] Arandjelovic B, Bogunovich D (2014). City profile: Berlin. Cities.

[ref-3] Backus-Freer J, Pyron M (2015). Concordance among fish and macroinvertebrate assemblages in streams of Indiana, USA. Hydrobiology.

[ref-4] Bagrowicz T, Fortuniak A, Górecki M, Lewandowska M, Ziułkiewicz M (2017). Hydrochemical transformations of river waters during the flow in the reception basin on the basis of Olechówka River in Łódź. E3S Web of Conferences.

[ref-5] Bohr R (1962). Socjologiczne badania peryfitonu roślinnego w jeziorze Mamry. (Sociological studies of the plant periphyton in Lake Mamry). St. Social Science.

[ref-6] CEMAGREF (1982). Etude des méthods biologiques quantitatives d’appréciation de la qualité des eaux. (Study on biological quantitative methods of water quality assessment). Rapport Division Qualite des Eaux Lyon-A.F. Pierre-Bénite, France, Bassin Rhône-Méditerranée-Corse (in French).

[ref-7] Charles DF, Kelly MG, Stevenson JR, Poikane S, Theroux S, Zgrundo A, Cantonati M (2021). Benthic algae assessments in the EU and the US: striving for consistency in the face of great ecological diversity. Ecological Indicators.

[ref-8] Cholnoky B (1968). Die Ökologie der Diatomeen in Binnengewässern (The ecology of diatoms in inland waters).

[ref-9] Cieślak-Arkuszewska A (2020). A River in small town landscape. Space & Form.

[ref-10] Clarke KR, Gorley RN, Somerfield PJ, Warwick RM (2014). Change in marine communities: an approach to statistical analysis and interpretation.

[ref-11] Coste M, Ayphassorho H (1991). Etude de la qualité des eaux du bassin Artois Picardie à l’aide des communautés de diatomé es benthiques (Application des indices diatomiques) France, Rapport Cemagref Bordeaux –Agence de l’Eau Artois Picardie. (in French).

[ref-12] Descy JP (1979). A new approach to water quality estimation using diatoms. Nova Hedwigia.

[ref-13] Dolédec S, Statzner B, Bournard M (1999). Species traits for future biomonitoring across ecoregions: patterns along a human-impacted river. Frehswater Biology.

[ref-14] Dumnicka E, Jelonek M, Kwandrans J, Wojtal A, Zurek R (2006). Ichtiofauna i status ekologiczny wód Wisły, Raby, Dunajca i Wisłoki (Ichthyofauna and ecological status of the waters of the Vistula, Raba, Dunajec and Wisłoka).

[ref-15] European Union (2000). Directive 2000/60/EC of the European Parliament and of the Council of 23 2000 establishing a framework for community action in the field of water policy. Official Journal of the European Communities L.

[ref-16] Fortuniak A, Hałas K, Górecki M, Szewczyk K, Wojtania A, Ziułkiewicz M (2018). Lifting of biogenic compounds by a riverbed from a municipal drainage basin on a day scale. E3S Web of Conferences.

[ref-17] Freedman JA, Lorson BD, Taylor RB, Carline RF, Stauffer JR (2014). River of the dammed: longitudinal changes in fish assemblages in response to dams. Hydrobiology.

[ref-18] Gillett ND, Oudsema ME, Steiman AD (2017). Live diatoms as indicators of urban stormwater runoff. Environmental Monitoring and Assessment.

[ref-19] Gillett ND, Pan Y, Manoylov KM, Stevenson JR (2011). The role of live diatoms in bioassessment: a large-scale study of Western US streams. Hydrobiologia.

[ref-20] Hofmann G (1994). Aufwuchs –Diatomeen in Seen und ihre Eignung als Indikatoren der Trophie [Proceedings - diatoms in lakes and their use as indicators of trophic state]. Bibliotheca Diatomologica.

[ref-21] Hofmann G, Werum M, Lange-Bertalot H (2011). Diatomeen im Sü *β*wasser-Benthos von Mitteleuropa. Rugell, Liechtenstein: A.R.G. Gantner Verlag K.G. (in German).

[ref-22] Holmes M, Taylor JC (2015). Diatoms as water quality indicators in the upper reaches of the Great Fish River, Estern Cape, South Africa. African Journal of Aquatic Science.

[ref-23] Houk V, Klee R, Tanaka H (2010; 2014). Atlas of freshwater centric diatoms with a brief key and descriptions. Part III. Stephanodiscaceae A, Cyclotella, Tertiarius, Discotella. Part IV. Stephanodiscaceae B, Stephanodicsus, Cyclostephanos, Pliocaenicus, Hemistephanos, Stephanocostis, Mesodictyon & Spicaticribra. Fottea 10, Supplement, 498 pp.; Fottea 14, Supplement, 529 pp.

[ref-24] Kahlert M, Ács E, Almeida SFP, Blanco S, Drezßler M, Ector L, Karjalainen SM, Liess A, Mertens A, vander Wal J, Vilaste S, Werner P (2016). Quality assurance of diatom counts in Europe: towards harmonized datasets. Hydrobiology.

[ref-25] Kelly MG, Gómez-Rodríguez C, Kahlert M, Almeida SFP, Bennett C, Bottin M, Vilbaste S (2012). Establishing expectations for pan-European diatom based ecological status assessments. Ecological Indicators.

[ref-26] Kelly MG, Juggins S, Guthrie R, Pritchard S, Jamieson J, Rippey B, Yallop M (2008). Assessment of ecological status in U.K. rivers using diatoms. Freshwater Biology.

[ref-27] Kelly MG, Whitton BA (1995). The trophic Diatom Index: a new index for monitoring eutrophication in rivers. Journal of Applied Phycology.

[ref-28] Kruk A, Ciepłucha M, Zięba G, Błońska D, Marszał L, Tybulczuk S, Penczak T (2017). Disturbed fish fauna zonation as an indicator of large-scale human impact: a case study (2011–2012) of the large, lowland Warta River, Poland. Journal of Applied Ichthyology.

[ref-29] Lange-Bertalot H, Hofmann G, Werum M, Cantonati M (2017). Freshwater benthic diatoms of central Europe: over 800 common species used in ecological assessment. Koeltz Botanical Books.

[ref-30] Latinopoulos D, Spiliotis M, Ntislidou Kagalou I, Bobori D, Tsiaoussi V, Lazaridou M (2021). One out–all out principle in the water framework directive 2000 - a new approach with fuzzy method on an example of greek lakes. Water.

[ref-31] Lavoie I, Campeau S, Zugic-Drakulic N, Winter J, Fortin C (2014). Using diatoms to monitor stream biological integrity in Eastern Canada: an overview of 10 years of index development and ongoing challenges. Science of the Total Environment.

[ref-32] Lenoir A, Coste M, Whitton BA, Rott E (1996). Development of a practical diatom index of overall water quality applicable to the French National Water Board network. Use of algae for monitoring rivers II.

[ref-33] Leukart P, Mollenhauer D (1997). Studies on algal drift in a small soft-water stream in the Spessart mountains. Germany. Nova Hedwigia.

[ref-34] Mackin L, Lewin J (2018). River stresses in anthropogenic times: large-scale global patterns and extended environmental timelines. Progress in Physical Geography.

[ref-35] Rakowska B (2001). Studium różnorodności okrzemek ekosystemów wodnych Polski niżowej (Study on the diatom diversity of the Polish lowland aquatic ecosystems).

[ref-36] Rimet F, Bouchez A (2012). Life-forms, cell-sizes and ecological quilds of diatoms in European rivers. Knowledge and Management of Aquatic Ecosystems.

[ref-37] Round FE, Crawford RM, Mann DG (1990). The Diatoms. Biology and morphology of the genera.

[ref-38] Stenger-Kovács C, Tóth L, Tóth F, Hajnal E, Padisák J (2014). Stream order-dependent diversity metrics of epilithic diatom assemblages. Hydrobiology.

[ref-39] Stevenson J (2014). Ecological assessments with algae: a review and synthesis. Journal of Phycology.

[ref-40] Stevenson RJ, Pan Y, Van Dam H, Smol JP, Stoermer EF (2010). Assessing environmental conditions in rivers and streams with diatoms. The Diatoms: applications for the Environmental and Earth Sciences.

[ref-41] Szczepocka E, Nowicka-Krawczyk P, Kruk A (2018). Deceptive ecological status of urban streams and rivers –evidence from diatom indices. Ecosphere.

[ref-42] Szczepocka E, Żelazna Wieczorek J (2018). Diatom biomonitoring –scientific foundations, commonly discussed issues and frequently made errors. Oceanological and Hydrobiological Studies.

[ref-43] Szczepocka E, Żelazna Wieczorek J, Nowicka-Krawczyk P (2019). Critical approach to diatom-based bioassessment of the regulated sections of urban flowing water ecosystems. Ecological Indicators.

[ref-44] Tszydel M, Markowski M, Majecki J (2016). Larvae of *Hydropsyche angustipennis* (Trichoptera, Hydropsychidae) as indicators of stream contamination by heavy metals in Łódź agglomeration. Zootaxa.

[ref-45] Tszydel M, Markowski M, Majecki J, Błońska D, Zieliński M (2015). Assessment of water quality in urban streams based on larvae of *Hydropsyche angustipennis* (Insecta, Trichoptera). Environmental Science and Pollution Research.

[ref-46] Van Dam H, Mertens A, Sinkeldam J (1994). A coded checklist and ecological indicator values of freshwater diatoms from the Nertherlands. Netherlands Journal of Aquatic Ecology.

[ref-47] Vannote RL, Wayne-Minshall G, Cummins KW, Sedell JR, Cushing CE (1980). The river continuum concept. Canadian Journal of Fisheries and Aquatic Sciences.

[ref-48] Winiwarter V, Haidvogl G, Hohensinner S, Hauer F, Bürkner M (2016). The long-term evolution of urban waters and their nineteenth century transformation in European cities. A comparative environmental history. Water History.

[ref-49] Żelazna-Wieczorek J, Nowicka-Krawczyk P (2015). The cascade construction of artificial ponds as a tool for urban stream restoration –the use of benthic diatoms to assess the effects of restoration practices. Science of the Total Environment.

[ref-50] Zgrundo A, Peszek Ł, Poradowska A (2018). Podręcznik do monitoringu i oceny rzecznych jednolitych części wód powierzchniowych na podstawie fitobentosu. (Handbook for the monitoring and assessment of river surface water bodies on the based of phytobenthos).

[ref-51] Ziułkiewicz M, Górecki M, Fortuniak A, Walas A, Grulke R (2019). 24-hour nutrient mass balance of small storage reservoir included in municipal rainwater drainage system. E3S Web of Conferences.

