# Supplementary material for: Response of diatom assemblages to the disruption of the running water continuum in urban areas, and its consequences on bioassessment
Source: PeerJ. 2021 Nov 23;9:e12457. doi: 10.7717/peerj.12457 (PMC8621708; doi:10.7717/peerj.12457)
Supplement: Supplemental Information 2 [file peerj-09-12457-s002.docx]

|  | Os1 | Os2 | Os3 | Os4 | Os5 |
| --- | --- | --- | --- | --- | --- |
| Water temp. [°C] | 2.7 – 16.5  9.2 | 1.7 – 23.0  12.4 | 2.5 – 19.0  12.0 | 3.0 – 18.0  10.6 | 4.0 – 17.0  10.6 |
| pH | 6.91 – 7.49  7.32 | 6.87 – 7.61  7.2 | 6.90 – 7.56  7.2 | 6.78 – 7.42  7.1 | 7.06 – 7.48  7.2 |
| EC [µS µScm^-1^] | 608 – 1115  888.2 | 197 – 814  413.3 | 236 – 1192  583 | 357 – 985  571 | 371 – 1200  647 |
| DOC [mgl^-1^] | 2.80 – 9.39  6.47 | 1.89 – 4.05  3.3 | 2.20 – 5.45  4.15 | 1.65 – 5.90  3.48 | 3.60 – 5.90  4.6 |
| BOD_5_  [mgl^-1^] | 1.6 – 6.0  2.88 | 4.4 – 14.0  9.7 | 5.0 – 13.3  8.5 | 3.8 – 9.0  5.6 | 4.0 – 8.0  6.0 |
| Total N [mgl^-1^] | 2.78 – 6.18  4.09 | 1.80 – 2.74  2.0 | 1.80 – 2.90  2.04 | 1.80 – 2.59  2.1 | 1.80 – 2.42  2.3 |
| N-NH_4_  [mgl^-1^] | 0.039 – 0.060  0.043 | 0.039 – 0.60  1.600 | 0.039 – 0.710  0.260 | 0.132 – 0.490  0.300 | 0.071 – 1.310  0.700 |
| N-NO_3_  [mgl^-1^] | 1.10 – 4.03  2.83 | 0.02 – 1.17  0.52 | 0.02 – 1.05  0.64 | 0.92 – 5.54  2.50 | 0.61 – 1.11  0.83 |
| Total P [mgl^-1^] | 0.10 – 0.16  0.13 | 0.10 – 0.26  0.16 | 0.10 – 0.29  0.15 | 0.10 – 0.17  0.12 | 0.10 – 0.19  0.15 |
| P-PO_4_  [mgl^-1^] | 0.09 – 0.10  0.08 | 0.03 – 0.10  0.08 | 0.03 – 0.10  0.08 | 0.03 – 0.26  0.13 | 0.03 – 0.40  0.20 |
